# Targeting of intracellular Ca^2+^ stores as a therapeutic strategy against age-related neurotoxicities

**DOI:** 10.1038/s41514-020-00048-1

**Published:** 2020-08-24

**Authors:** Joshua Goldberg, Antonio Currais, Gamze Ates, Ling Huang, Maxim Shokhirev, Pamela Maher, David Schubert

**Affiliations:** The Salk Institute for Biological Studies, 10010 North Torrey Pines Road, La Jolla, CA 92037 USA

**Keywords:** Alzheimer's disease, Alzheimer's disease

## Abstract

Calcium dysregulation often underlies pathologies associated with aging and age-associated neurodegenerative diseases. Cells express a unique pattern of Ca^2+^ channels and pumps geared to fulfill specific physiological requirements and there is a decline in the fidelity of these processes with age and age-associated diseases. J147 is an Alzheimer’s disease (AD) drug candidate that was identified using a phenotypic screening platform based upon age-related brain toxicities that are mediated by changes in calcium metabolism. The molecular target for J147 is the α-F1-ATP synthase (ATP5A). J147 has therapeutic efficacy in multiple mouse models of AD and accelerated aging and extends life span in flies. A bioinformatics analysis of gene expression in rapidly aging SAMP8 mice during the last quadrant of their life span shows that J147 has a significant effect on ion transport pathways that are changed with aging, making their expression look more like that of younger animals. The molecular basis of these changes was then investigated in cell culture neurotoxicity assays that were the primary screen in the development of J147. Here we show that J147 and its molecular target, ATP synthase, regulate the maintenance of store-operated calcium entry (SOCE) and cell death during acute neurotoxicity.

## Introduction

Calcium ions (Ca^2+^) impact most aspects of cell behavior and metabolism and their aberrant regulation is often associated with aging, Alzheimer’s disease (AD), and neurotoxicity^1,2^. The intracellular Ca^2+^ concentration ([Ca^2+^]) varies depending on its subcellular location and the maintenance of these Ca^2+^ stores is critical for the propagation of inter-organelle Ca^2+^ signals, such as those driving mitochondrial oxidative metabolism^3^ and the initiation of ER stress responses^4^. However, perturbations in Ca^2+^ mobilization can flood the cytoplasm with excess Ca^2+^, ultimately leading to cell death^5^. Therefore, a treatment to alleviate this form of acute, Ca^2+^-dependent toxicity would have significant therapeutic value.

The AD drug candidate J147 was identified using a phenotypic screening platform designed to select drug candidates that protect against toxicities associated with brain aging, some of which involve dysregulated Ca^2+^ homeostasis^6^. We have previously demonstrated J147’s therapeutic efficacy in mouse models of AD^7–9^ and accelerated aging^10–12^. The molecular target of J147 is the α-F1 mitochondrial ATP synthase (ATP5A)^11^, a central player in Ca^2+^ metabolism that can drive Ca^2+^ flux via changes in the ATP-generated H^+^ gradient or opening of the mitochondrial permeability transition pore (mPTP).

Here, we show that J147 reduces age-induced changes in the transcriptome of genes associated with Ca^2+^-related transport pathways in aged SAMP8 mice. Additionally, we demonstrate that both J147 and its molecular target, ATP synthase, affect Ca^2+^ mobilization across the cytoplasm, ER, and mitochondrial compartments following acute old age-associated toxic insults by its regulation of store-operated Ca^2+^ entry (SOCE).

## Results

### J147 modulates Ca^2+^ metabolism in vivo

Rapidly aging SAMP8 mice recapitulate much of the progressive, age-associated decline in brain function that is associated with the development of AD in humans, developing both cognitive deficits and pathological hallmarks of dementia by 9 months of age^13^. Changes in hippocampal gene expression were examined between 9-month-old control mice and 13-month-old mice fed either control diet or J147 for 4 months starting at 9 months of age, a treatment that suppresses the aging phenotype^12^. Because our screening platform is based in part upon calcium dysregulation, we used gene set enrichment analysis (GSEA) to ask how J147 affected age-associated changes corresponding to Ca^2+^-related ion channels and transporters.

**Fig. 1:**
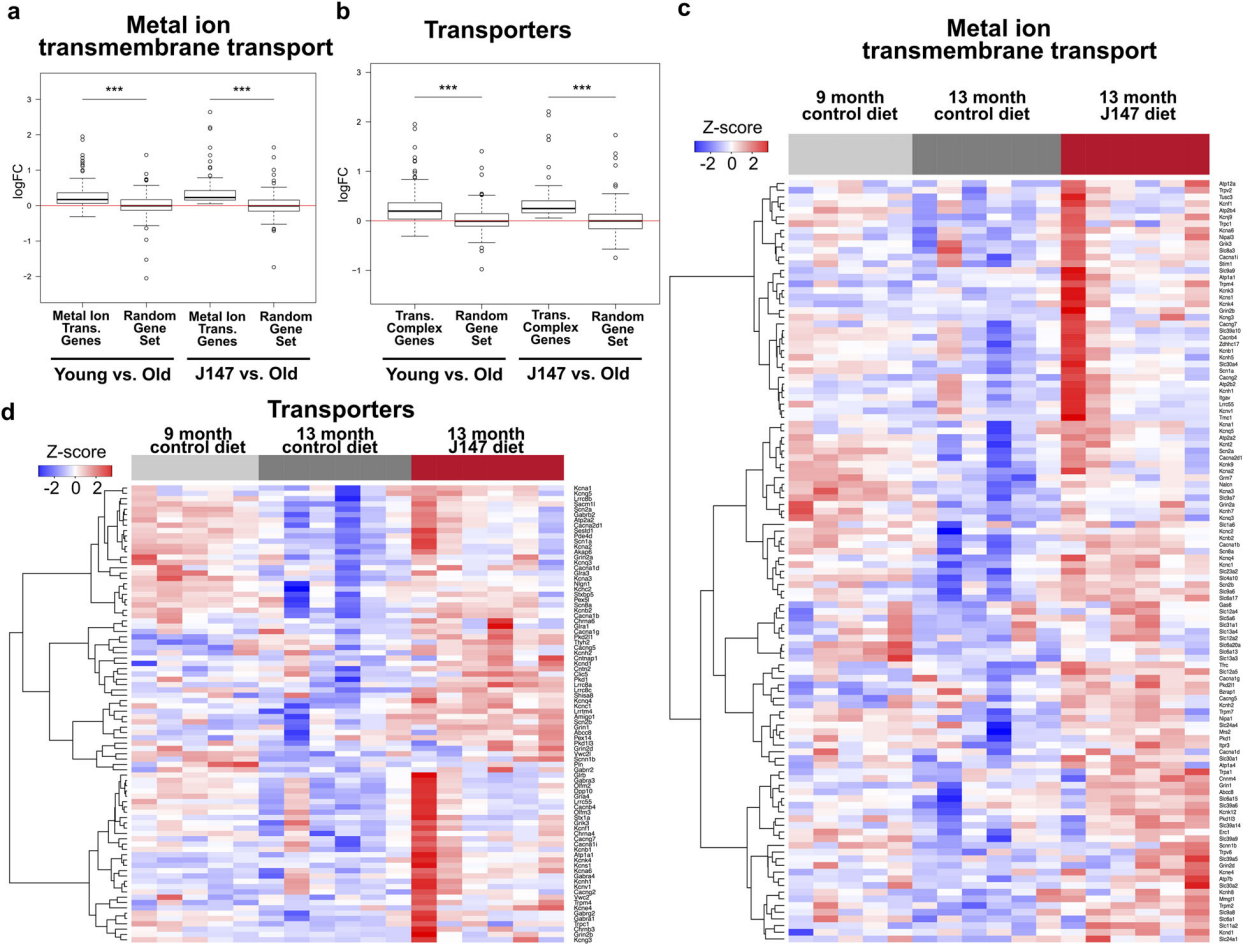
J147 prevents age-induced transcriptomic changes in Ca^2+^-related signaling pathways in SAMP8 mouse hippocampus. Boxplot of estimated expression fold changes for the leading edge genes as defined by GSEA for (**a**) metal ion transporter genes and (**b**) transporter complex genes. Equivalent numbers of randomly sampled genes from the genome were used as controls. Boxplot center line represents the median, box lower bounds and upper bounds represent the 25th and 75th percentile of the dataset, respectively. Box whiskers reach to data points that are no more than 1.5-fold of the interquartile range of the box*.* Wilcoxon rank sum test *** *p* value < 0.001. **c**, **d** Corresponding heatmaps of log-transformed gene expression for the leading edge genes. Red = high, blue = low. Treatment groups color-coded at bottom of heatmap; (light gray) left: 9-month control diet (five animals), (dark gray) middle: 13-month control diet (six animals), (red) right: 13-month + J147 diet (six animals).

There were significant, age-dependent differences in the expression of genes in these pathways that occurred between 9- and 13-months (Fig. 1a–d). Among the most affected genes were those involved in ER Ca^2+^ mobilization and SOCE, a specialized form of extracellular-derived Ca^2+^ influx, including the ATPase sarcoplasmic/endoplasmic reticulum Ca^2+^ transporter (Atp2a2), stromal interaction molecule 1 (Stim1), and inositol 1,4,5-triphosphate receptor type 3. Significant changes were also detected in genes residing in a variety of subcellular locations, including the plasma membrane, endosomes, golgi apparatus, mitochondria, and those involved in extracellular secretion (Supplementary Table 1). J147 prevented many of the age-induced changes in ion and Ca^2+^ transporter gene expression seen in control 13-month-old SAMP8 mice, normalizing them to levels found in the 9-month-old controls (Fig. 1c, d).

**Fig. 2:**
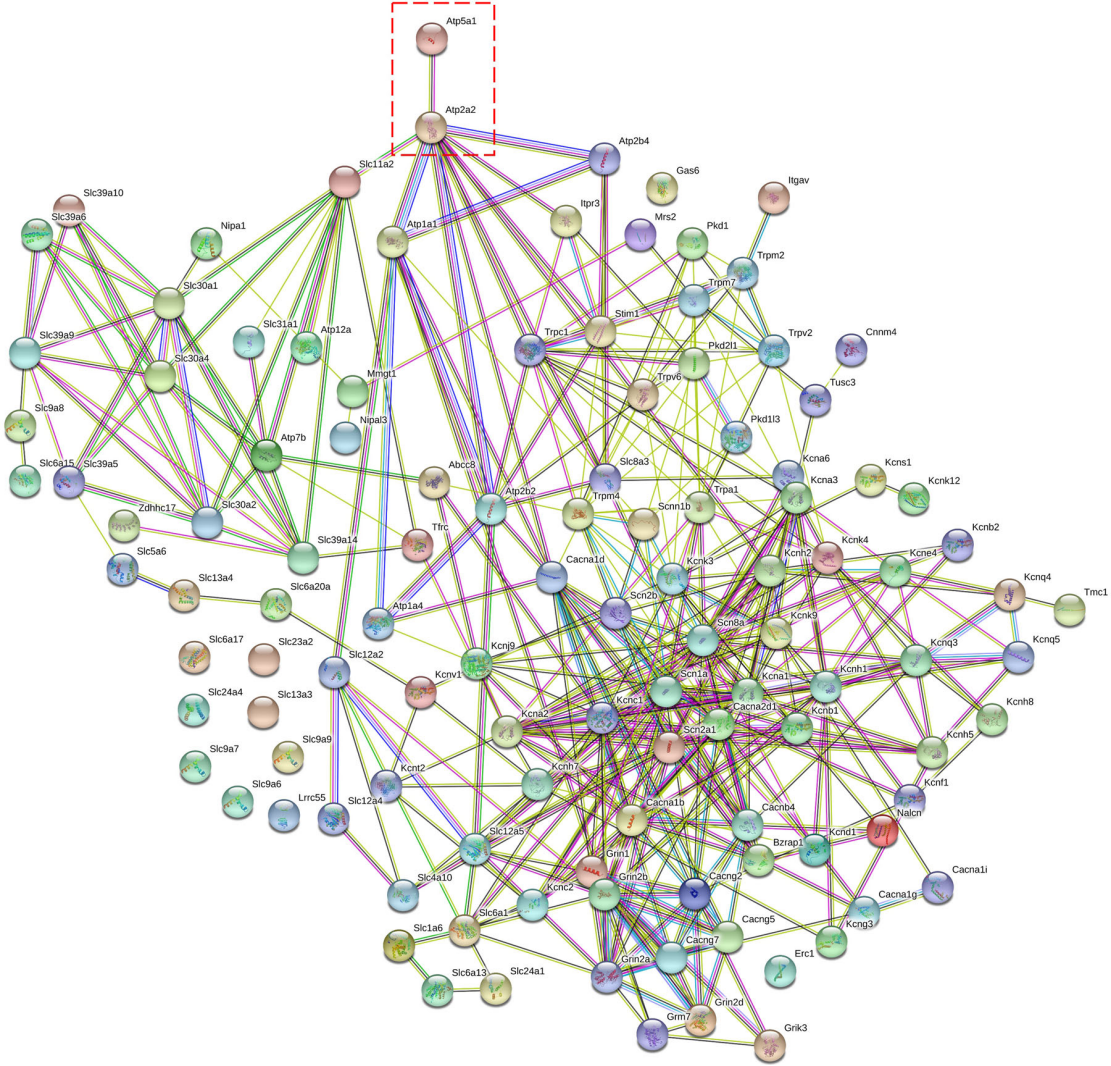
Network analysis based on the gene list. Metal ion trans-membrane transporter activity. Atp5a1 was added to the gene list as it is the known target of J147. Atp5a1 interacts with Atp2a2 (red box), implying a possible functional link between both genes based on murine co-expression.

Because the mitochondrial ATP synthase is the molecular target of J147, we incorporated protein–protein association networks onto our gene list with the addition of ATP synthase, and observed a specific interaction between ATP synthase and ATP2a2, the ER-resident sarco/endoplasmic reticulum Ca^2+^-ATPase (SERCA) pump responsible for controlling extracellular-derived Ca^2+^ influx. This suggests that ATP synthase, and mitochondria in general, can influence ER-derived Ca^2+^ signaling nodes (Fig. 2). These results led to the hypothesis that the neuroprotective effect of J147 in the acute neurotoxicity assays used for its development may be due to modulating SOCE. We therefore asked how J147 affects intracellular Ca^2+^ levels in the mitochondria and the ER, major sources of intracellular Ca^2+^ that play important roles in Ca^2+^ buffering.

### J147 alters Ca^2+^ homeostasis

**Fig. 3:**
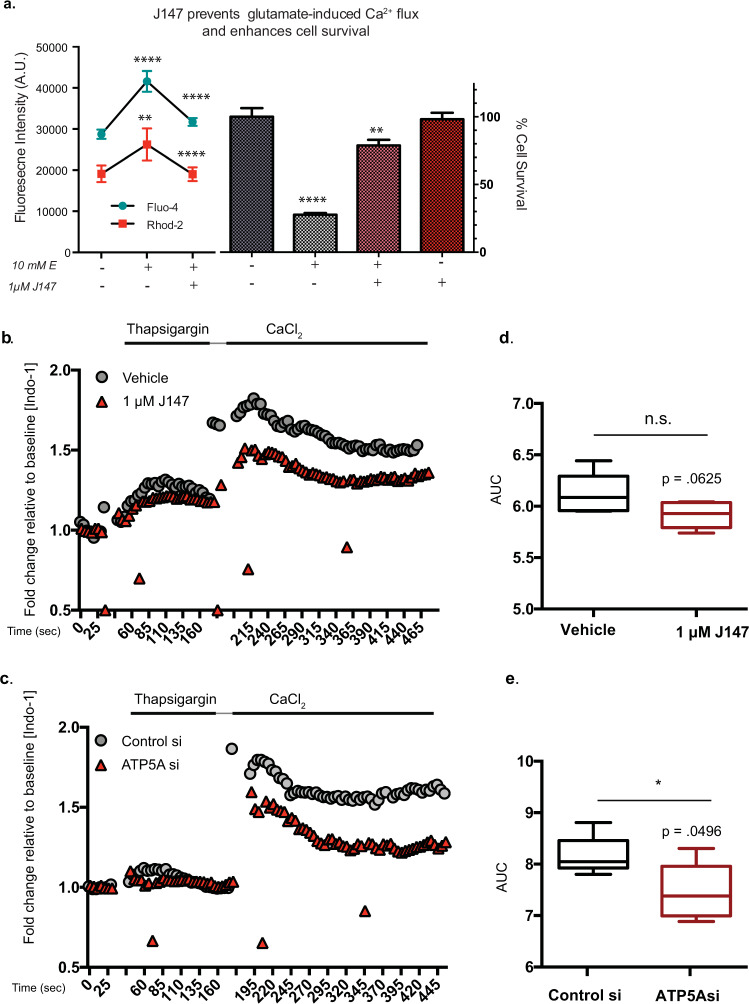
J147 dampens ionophore-induced Ca^2+^ influx in HT22 cells and SOCE. **a** (left) Overnight treatment with 10 mM glutamate (E) and 1 μM J147 using Fluo-4 (blue) and Rhod-2 (red). One-way ANOVA, Tukey’s multiple comparison, fluo-4 df = 27, rhod-2 df = 25. **p < 0.01 ****p < 0.0001. (right): MTT cell survival. One-way ANOVA, multiple comparisons (*n* = 3 exp., df = 8. ****p < 0.0001, **p < 0.01). **b**, **c** Indo-1 ratiometric cytosolic Ca^2+^ indicator dye was used in combination with flow cytometry-based kinetic flux analysis to assess SOCE in HT22 cells using a Ca^2+^ re-addition method^21^. After 1 h pretreatment with 1 μM J147, ER stores were depleted with 3 μM thapsigargin in Ca^2+^-free medium, after which time 3 mM CaCl_2_ was added to allow extracellular-derived cytosolic Ca^2+^ influx and measurement of SOCE. Data are representative of a single trace. Fold changes are normalized to baseline Indo-1 fluorescence for each treatment (**b**) or ATP5A siRNA-mediated knockdown (**c**). Area under the curve shown for *t* = 195–315 s for both conditions (**d**, **e**) encompassing the initial Ca^2+^ peak. Unpaired *t*-test Welch’s correction *(n* = 3 exp., *t* = 2.362, df = 7.124, *p < 0.05). The following values correspond to center line, upper/lower bounds, and whisker max/min for vehicle and J147, respectively: center line: 6.117,5.917; upper/lower bounds: 6.292, 6.035/5.958, 5.794; whiskers max/min: 6.443, 6.043/5.953, 5.738. The following values correspond to center line, upper/lower bounds, and whisker max/min for control and ATP5A si, respectively: center line: 8.139, 7.452; upper/lower bounds: 8.869, 7.799/7.605, 7.115; whiskers max/min: 9.24, 8.362/6.902, 5.934.

Ca^2+^ influx is required for execution during a regulated cell death pathway that we characterized and named oxytosis in 2001^14^. This pathway is identical to ferroptosis^15^, and henceforth is called oxytosis/ferroptosis. Because this cell death pathway recapitulates many aspects of nerve cell death seen in the aging brain^15^, it was used as the primary neurotoxicity screen in our drug discovery platform^6^. In oxytosis, high doses of extracellular glutamate inhibit the glutamate/cystine antiporter system x_c_^−^. The decrease in cystine import reduces the amount of intracellular cysteine, the rate-limiting amino acid in glutathione (GSH) synthesis, resulting in GSH loss and detrimental increases in reactive oxygen species (ROS) and subsequently Ca^2+^ influx and cell death^14,16,17^. Although ROS accumulation initiates Ca^2+^ influx, Ca^2+^ influx also further enhances ROS production^14^. Importantly, Ca^2+^ influx is required for cell death^14,17^. Thus, we determined the effect of J147 on Ca^2+^ levels in the cytosolic and mitochondrial compartments after overnight treatment with glutamate. Figure 3a shows that glutamate (E) treatment significantly increased both [Ca^2+^]_cyt_ and [Ca^2+^]_mit_ in HT22 nerve cells, as measured by Fluo-4 and Rhod-2 Ca^2+^ indicator dyes specific to the cytoplasm and mitochondria, respectively. J147 not only enhanced cell survival, but also dampened the late-stage cytoplasmic and mitochondrial Ca^2+^ influx.

### J147 and ATP synthase regulate store-operated Ca^2+^ entry (SOCE)

The maintenance of SOCE is critical for the survival of cells. SOCE-mediated toxicity is triggered when [Ca^2+^]_er_ depletion leads to the translocation of the ER membrane-resident protein Stim1/2 to the plasma membrane where recruitment of calcium release-activated calcium modulator (Orai) Ca^2+^ channel proteins promote extracellular-derived Ca^2+^ influx into the cytosol to replenish ER stores. Dysregulation of SOCE is observed in both aging and AD^18^. The interaction of ATP synthase and ATP2a2 uncovered through our protein–protein network analysis supports recent reports demonstrating the direct involvement of SOCE proteins in oxytosis/ferroptosis^19,20^. Therefore, we asked whether J147 and modulation of ATP synthase activity via siRNA-mediated knockdown, affect SOCE.

SOCE kinetics were measured via flow cytometry using a Ca^2+^ re-addition method^21^ that allowed separation of the two phases of Ca^2+^ mobilization—ER Ca^2+^ release and extracellular Ca^2+^ influx. The fluorescent ratiometric Indo-1 Ca^2+^ indicator dye was used to monitor changes in [Ca^2+^]_cyt_ resulting from ER Ca^2+^ release and extracellular-derived influx. First, baseline recordings of [Ca^2+^]_cyt_ were performed in the absence of extracellular Ca^2+^. This was followed by the addition of 3 μM thapsigargin to empty ER Ca^2+^ stores into the cytosol, after which 3 mM CaCl_2_ was added back to the culture medium to allow for extracellular-derived Ca^2+^ influx. Figure 3b–e shows that despite having a minimal effect on the cytosolic Ca^2+^ increase during the first phase of ER depletion, both J147 and ATP5A knockdown still dampen the extracellular-derived Ca^2+^ influx in HT22 cells. This suggests that ATP synthase and by extension mitochondria exert control over an ER-regulated Ca^2+^ influx pathway involving the regulation of extracellular-derived Ca^2+^ influx that is dampened by J147 and ATP synthase knockdown.

Thus, alterations in Ca^2+^ buffering between the ER and mitochondria may underlie the abilities of J147 and reduced ATP synthase activity to prevent detrimental Ca^2+^ overload.

### J147 and modulation of ATP synthase activity buffer against ER Ca^2+^ release and transfer to mitochondria

The ER and the mitochondria serve as intracellular Ca^2+^ stores that, when used for buffering, can modulate Ca^2+^-derived signaling events. Ca^2+^ transfer between the ER and mitochondria can serve as a way to regulate these Ca^2+^-derived signals^22^. Up to this point, our data suggest that SOCE can be regulated by the mitochondria as J147 and ATP5A knockdown both dampen SOCE. Therefore, we asked if J147 and ATP synthase had specific effects on inter-organelle Ca^2+^ transfer between the ER and mitochondria.

**Fig. 4:**
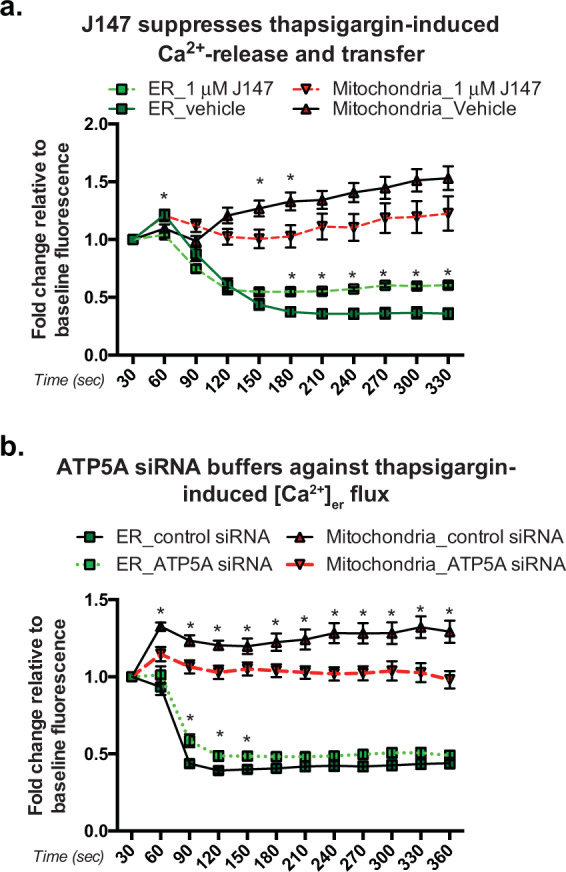
J147 and ATP synthase knockdown buffer against ER Ca^2+^ release and transfer to mitochondria. HT22 cells treated with 1 μM J147 or ATP5A siRNA transfection were co-transfected with ER and mitochondrial-specific fluorescent Ca^2+^ sensors to monitor changes in fluorescence via *Z*-stack confocal microscopy live-cell imaging (**a**) after 5 μM thapsigargin treatment in Ca^2+^-free media. Baseline fluorescence was recorded for 30 s before addition of thapsigargin. Fold changes in fluorescence for J147 (**a**) and ATP5A siRNA (**b**) were recorded at 30 s intervals and normalized to baseline fluorescence intensity for each condition in each treatment group. The Ca^2+^ sensors are ER (green) and mitochondria (red). Multiple *t*-test (Holm–Sidak method); *n* = 21 and 25 cells examined in **a** and **b**, respectively. *p < 0.05; scale bar = 10 μM.

To address this, we co-expressed genetically encoded fluorescent Ca^2+^ sensors specific to the ER and mitochondria in HT22 cells and performed three-dimensional live-cell imaging to monitor changes in fluorescence in real time (Fig. 4). Cells were preincubated with either vehicle or J147 for 24 h in Ca^2+^-replete medium, after which the medium was replaced with Ca^2+^-free medium immediately prior to recording. Baseline Ca^2+^ levels were recorded for 30 s, followed by the addition of thapsigargin to deplete ER Ca^2+^. The *Z*-stack images are shown in Supplementary Fig. 1.

The inhibition of ER-resident SERCA pumps with thapsigargin revealed a J147-specific effect on ER Ca^2+^ retention. As expected, inhibition of SERCA led to a decrease in [Ca^2+^]_er_ (Fig. 4a; green symbols, solid line and increased [Ca^2+^]_mit_ (red symbols, solid line). J147 dampened the extent of Ca^2+^ release from the ER (green symbols, dotted lines), and thus reduced the subsequent rise in mitochondrial Ca^2+^ observed in vehicle-treated cells (red symbols, dotted lines).

To confirm the target specificity of J147 and its role in Ca^2+^ metabolism, knockdown of ATP5A phenocopied the suppression of ER Ca^2+^ release during 5 μM thapsigargin treatment (Fig. 4b; green symbols, solid vs. dotted lines). Additionally, the increase in mitochondrial Ca^2+^ after thapsigargin treatment was also decreased in ATP5A knockdown cells (red symbols, solid vs. dotted lines). These data show that J147 has an ER-specific effect on Ca^2+^ maintenance, and point to mitochondrial involvement in the maintenance of ER Ca^2+^ stores as a way to regulate SOCE.

### J147 prevents Ca^2+^ dysregulation in multiple age-associated neurotoxicities

**Fig. 5:**
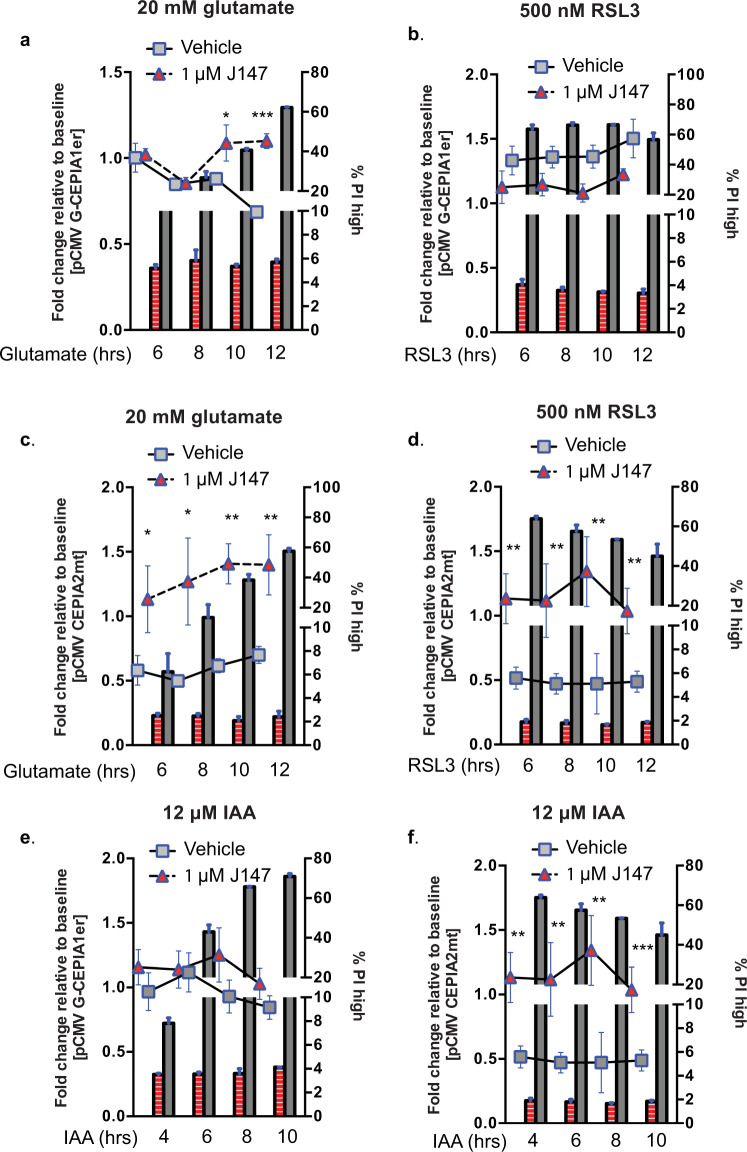
J147 prevents/normalizes Ca^2+^ fluctuations during glutamate, RSL3, and IAA toxicity. Flow cytometry was used to assess Ca^2+^ changes in propidium iodide (PI) positive HT22 cells transfected with genetically encoded Ca^2+^ sensors targeted to the ER or mitochondria (red (J147) and gray (vehicle) symbols, dotted lines, left *y*-axis). Propidium iodide (PI) was used to separate cell populations corresponding to cell viability (red (J147) and gray (vehicle) bars, right *y*-axis). 1 μM J147 was used in all conditions. ER (**a**, **b**) and mitochondria (**c**, **d**) Ca^2+^ levels were measured after the addition of 10 mM glutamate or 500 nM RSL3. ER (**e**) and mitochondria (**f**) Ca^2+^ levels were measured after the addition of 12 μM IAA. Fold changes were normalized to the basal fluorescence of vehicle untreated cells. Data are plotted as geometric mean fluorescence intensity fold change, 20,000–30,000 cells analyzed per time point. *n* = 3 exp. for **a**–**f**. Multiple *t*-test (Holm–Sidak method),*p < 0.05, **p < 0.01, ***p < 0.001.

Neurodegenerative diseases are often the result of combined cellular stresses. Because J147 suppresses ER Ca^2+^ release and thus affects [Ca^2+^]_mit_ and ER–mitochondrial Ca^2+^ transfer in cells treated with glutamate (Fig. 5a, c), we asked if J147 could protect HT22 cells from similar alterations in Ca^2+^ mobilization when the cells were subjected to additional neurotoxicities.

RSL3-mediated inhibition of GSH peroxidase 4 activates the oxytosis/ferroptosis pathway downstream of GSH loss^23^. To determine if different inducers of oxytosis/ferroptosis have similar effects on Ca^2+^ levels, and if J147 can normalize these changes, HT22 cells were treated with glutamate or RSL3 alone or in the presence of J147 and monitored for fluorescent changes across ER and mitochondrial compartments over time. J147 blocked the glutamate and RSL3-induced decreases in cell viability (percent PI^high^ population) (Fig. 5a–d). Upon examination of the compartmental changes in Ca^2+^ in the ER and mitochondria, J147 had only modest effects on the ER responses (Fig. 5a, b). In contrast, consistent decreases in [Ca^2+^]_mit_ were observed in the PI^high^ population in response to both insults which correlated with the loss of cell viability (Fig. 5c, d). Importantly, these changes were reduced by J147 treatment (Fig. 5c, d). Thus, the targeting of ER–mitochondrial Ca^2+^ by J147 contributes to its neuroprotective effects against both glutamate and RSL3, further supporting data showing that oxytosis and ferroptosis are the same^15^.

**Fig. 6:**
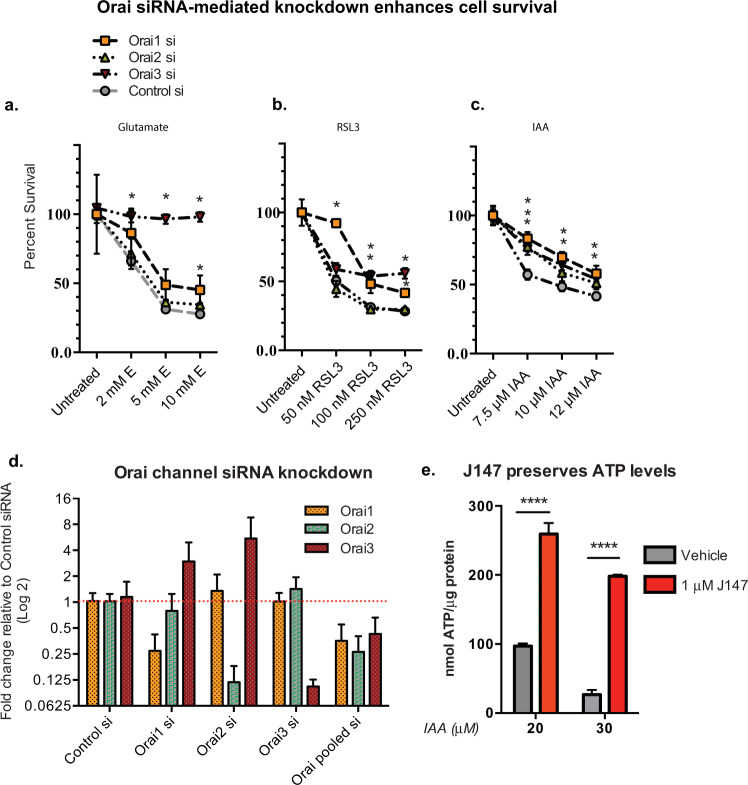
Orai siRNA-mediated knockdowns improve cell survival. HT22 cells were transfected with siRNA to Orai 1, 2, or 3 and assessed for cell viability after 24 h treatment with **a** 10 mM glutamate, **b** 250 nM RSL3, or **c** 12 μM IAA (*n* = 3 exp., multiple *t*-test (Holm–Sidak method); df = 4; *p < 0.05). **d** qPCR validation of Orai channel knockdown. **e** 1 μM J147 preserves ATP levels during 20 and 30 μM IAA treatment. *p* < 0.05 (*n* = 3 exp., unpaired *t*-test).

Decreases in energy levels often accompany old age and age-related neurodegeneration^24^. This can be mimicked in vitro by treating cells with iodoacetic acid (IAA), an irreversible inhibitor of glyceraldehyde-3-phosphate dehydrogenase, resulting in inhibition of glycolysis and cell death in HT22 cells^11^. Similar to the effects observed in glutamate and RSL3 toxicities, IAA had a minimal effect on ER Ca^2+^ (Fig. 5e), but caused a decrease in mitochondrial Ca^2+^ levels that was prevented along with the loss in cell viability by J147 (Fig. 5f). Since reducing potential is tightly linked to ATP production and energy homeostasis, it was asked if J147’s ability to suppress negative changes in mitochondrial Ca^2+^ levels may be a reflection of its ability to maintain ATP levels during IAA toxicity. Figure 6e shows that J147 maintained ATP at a high level suggesting that J147 increases mitochondrial ATP synthesis. Taken together, our data show that dysregulated mitochondrial Ca^2+^ flux is a critical factor underlying several forms of nerve cell toxicity and that J147 enhances cell viability by preserving mitochondrial Ca^2+^.

### Calcium activated release calcium modulator (Orai) channels contribute to cell death

J147 and ATP synthase knockdown dampen SOCE and prevent cell death, suggesting that modulation of extracellular-derived Ca^2+^ influx is critical for cell death. To test this idea, we knocked down the three plasma membrane Ca^2+^-channel proteins responsible for mediating the extracellular-derived Ca^2+^ influx phase of SOCE, Orai 1, 2, and 3 and determined if this could modulate late-stage cell death induced by treatment with glutamate, RSL3, and IAA. Orai3 knockdown completely protected against glutamate toxicity, while Orai1 and Orai3 knockdown were both effective against RSL3 toxicity, albeit knockdown of Orai1 was more effective at 50 nM RSL3 (Fig. 6a, b). In contrast, knockdowns of all three Orai channels significantly enhanced cell survival in IAA-induced toxicity (Fig. 6c). While the extent of protection afforded by each Orai channel knockdown varied, Orai3 knockdown consistently provided the best protection at the highest concentrations of glutamate, RSL3, and IAA tested. Interestingly, knockdown of both Orai1 and Orai2 upregulated the levels of Orai3 mRNA (Fig. 6d) suggesting that channels comprised of Orai3 are critical in regulating Ca^2+^-flux in these neurotoxicities. These data demonstrate that Orai channels and SOCE are involved in cell death in neurotoxicity paradigms that mimic various aspects of age-associated decline.

## Discussion

J147 was selected as a lead compound for the treatment of AD due to its neuroprotective effects in our phenotypic drug screening platform designed to mimic a core group of age-associated brain toxicities that together contribute to the development of AD^6,9^. Since then, we have demonstrated J147’s efficacy in multiple mouse models of AD and cognitive decline^7,8,10,12^ and identified its molecular target as the α-F1 mitochondrial ATP synthase^11^. The fact that our primary neurotoxicity screening assay in HT22 cells is based upon the oxytosis/ferroptosis pathway that involves alterations in Ca^2+^ homeostasis led us to investigate how J147 and its target can affect intracellular Ca^2+^ mobilization.

Increased Ca^2+^ release from IP3R and ryanodine receptors (RyR) occurs in aged neurons, contributing to significant increases in ER–mitochondrial transfer of Ca^2+^^25^. Similarly, age-related changes in the expression and/or activity of Ca^2+^-release/uptake channels have also been reported^26^. In fact, hippocampal overexpression of FK506-binding protein 12.6/1b (*FKBP1b*), a negative regulator of RyR Ca^2+^ release, reverses aging-induced memory impairment, and neuronal Ca^2+^ dysregulation in rats^27^. *FKBP1b* overexpression also counteracted expression changes in 37% of age-dependent genes, emphasizing a vast transcriptional network being affected by the disruption of Ca^2+^ homeostasis^27^.

Initially, we asked if genes involved in Ca^2+^ metabolism were associated with aging and the development of dementia in the SAMP8 accelerated aging mouse model. RNAseq analysis revealed a specific, age-induced dysregulation of signaling pathways involved in Ca^2+^ and ion transporters and channels including proteins that regulate Ca^2+^-flux into and out of the ER during SOCE. Importantly, these changes were prevented by J147 when fed to old, symptomatic mice. By combining a protein–protein interaction network with gene expression data, we uncovered an interaction between ATP synthase and the ER SERCA pump, ATP2a2, implicating ATP synthase involvement in SOCE. We then asked if J147 and ATP synthase knockdown could affect SOCE in cultured nerve cells. Indeed, both dampened SOCE. While ATP synthase has been implicated in Ca^2+^-efflux from the mitochondria during mPTP formation during cell death, to our knowledge a direct role for ATP synthase in moderating SOCE has not been previously established. Furthermore, this suggests that dampening SOCE is involved in the neuroprotective effects of J147.

Specialized ER–mitochondrial contacts called mitochondrial–ER associated membranes (MAMs) facilitate Ca^2+^ exchange between these two organelles. While controlled levels of ER–mitochondrial Ca^2+^ flux are crucial for driving metabolism^28^, excessive IP_3_R3-mediated ER Ca^2+^ release can lead to mitochondrial Ca^2+^ overload, opening of the mPTP, cytochrome *c* release, caspase activation, and apoptotic cell death^29^. Thus, any alteration in this interface is expected to have pleiotropic effects on cell homeostasis and survival. Therefore, we examined what effect J147 and modulation of ATP synthase activity had on intracellular Ca^2+^ store maintenance and transfer between the ER and mitochondria by treating cells with thapsigargin to assess intracellular store release. Thapsigargin treatment increased [Ca^2+^]_mit_, demonstrating SERCA pump inhibition can increase mitochondrial Ca^2+^ uptake. J147 and ATP5A knockdown increased retention of ER Ca^2+^ and minimized mitochondrial influx, indicating that a dampening of ER–mitochondrial Ca^2+^ transfer is a contributing factor in neuroprotection by J147.

Our data also show that distinct neurotoxic insults have similar effects on mitochondrial Ca^2+^ maintenance, suggesting a conserved Ca^2+^-mediated cell death pathway involving mitochondrial control over SOCE. Oxytosis/ferroptosis can be induced by glutamate and RSL3, acting at different sites in the cell death pathway. We assessed changes in ER and mitochondrial Ca^2+^ following glutamate or RSL3 treatment and observed decreases in mitochondrial Ca^2+^ during both, thus demonstrating that oxytosis and ferroptosis overlap in their convergence on mitochondrial Ca^2+^ dysregulation. Similarly, energy deprivation via the inhibition of glycolysis induced a decrease in [Ca^2+^]_mit_. J147 prevented Ca^2+^ release from the mitochondria in response to all three insults and prevented the loss of ATP with reduced glycolysis.

To confirm the contribution of SOCE to cell death induced by the three insults, we knocked down the major plasma membrane Orai pore forming proteins that mediate SOCE influx. Interestingly, knockdown of different Orai channels elicited varying levels of protection depending on the toxic insult. Although there is an Orai channel isoform-specific cell survival enhancement for each neurotoxicity model, Orai3 knockdown was most effective overall. Furthermore, increased levels of Orai3 mRNA were observed in both Orai1 and Orai2 knockdown cells, emphasizing its importance among Orai channel subtypes. These data argue that components of SOCE represent a conserved Ca^2+^-dependent cell death pathway that is a potential drug target for neurotoxic insults associated with aging and disease.

In summary, gene expression analysis revealed that a dysregulation in Ca^2+^-associated pathways occurs during aging, and that administration of J147 to old mice prevents many of these changes. J147 and modulation of its molecular target, ATP synthase, stabilize mitochondrial Ca^2+^ levels, and minimize SOCE activity to protect cells from multiple, acute neurotoxicities. Since the ER and mitochondria are critical organelles that utilize Ca^2+^ to drive metabolism and stress responses, identifying drug candidates similar to J147 that target underlying Ca^2+^-mediated toxicities may be a strategy to combat both aging and age-related neurodegeneration.

## Methods

### Cell viability and acute toxicity

Mouse hippocampal HT22 neuronal cells were propagated in DMEM/high glucose with 10% fetal calf serum as previously described^30^. Cell viability was determined by MTT assays in 96-well plates^9^ and propidium iodide (PI) (Thermofisher; P3566) uptake during flow cytometry. Oxytosis and IAA toxicity were performed as previously reported^9^. HT22 cell survival responses to RSL3 (Sigma; 2234) toxicity were measured and performed identically to the oxytosis and IAA toxicity assays. For all of the toxicity assays, HT22 cells were plated at 5 × 10^3^ in 96-well plates, grown overnight, and then treated with the indicated concentrations of the toxins for the indicated times at which point cell death was measured by the MTT assay or PI uptake as indicated in the figure legends. All siRNAs were acquired from Santa Cruz Biotechnology. In experiments assessing the contribution of Orai-Ca^2+^ channel or ATP synthase proteins in the aforementioned toxicity assays, cells were transfected with siRNA (Santa Cruz Biotechnology) to either Orai1 (sc-76002), Orai2 (sc-,76004), Orai3 (sc-76006) or ATP synthase (sc-60228) for 24 h prior to seeding into 96-well plates followed by the MTT assay to assess cell vulnerability as previously described^10^.

### SAMP8 mouse, RNAseq, and bioinformatics analysis

The SAMP8 line was acquired from Harlan Laboratories (UK). Tissue preparation and collection were performed as previously described^10,12^. All experiments were performed in accordance with the US Public Health Service Guide for Care and Use of Laboratory Animals and the protocols were approved by the IACUC at the Salk Institute. Twenty-three 9-month-old female SAMP8 mice were fed with vehicle diet (LabDiet 5015, TestDiet, Richmond, IN), and 22 9-month-old female SAMP8 mice were fed with J147 (LabDiet 5015 + 200ppm J147, TestDiet, ~10 mg/kg/day). Diet treatment lasted for four months. This dose of J147 was shown to be effective in multiple mouse models of memory, AD and aging^8–10,12^. RNAseq, gene expression, and network analysis was performed as described^12^. Briefly, SAMP8 count data was re-analyzed by DESeq2 to estimate gene expression changes in different samples. GSEA was performed using WebGestaltR with genes ranked by their -log10(*p* value) times direction of expression changes (positive for upregulated genes and negative for down-regulated genes). Pathways with adjusted *p* value < 0.05 were identified as significantly altered (1000 permutation by gene). Log-transformed FPKM values of leading edge genes were used to generate the heatmap using package gplots.

### Ca^2+^ measurements

All Ca^2+^ indicator dyes were acquired from Thermofisher Scientific. Fluo-4 (excitation 488 nm (F14201)) and Rhod-2 (excitation/emission 552/581 nm (R1244) (AM, cell permeant forms)) Ca^2+^-indicator dyes specific to cytosol and mitochondria, respectively, were exclusively used in a 96-well plate format as previously described^11^ unless otherwise indicated. Briefly, HT22 cells were plated at 5 × 10^3^ in 96-well plates, grown overnight, and then treated as indicated in the figure legends. The dyes were then added to the cells for 45 min and the fluorescence was measured on a Spectramax M5 plate reader. Indo-1 (I1223) ratiometric Ca^2+^ indicator dye was used to measure cytosolic Ca^2+^ during flow cytometry experiments in accordance with the manufacturer’s protocol. Indo-1 maximum emission shifts from ~475 nm (Ca^2+^-unbound form) to ~400 nm (Ca^2+^-bound form). All fluorescent Ca^2+^ sensor plasmids were acquired from Addgene—ER (pCMV G-CEPIA1er, plasmid # 58215) and mitochondria (pCMV CEPIA2mt, plasmid # 58218; CMV-mito-LAR-GECO1.2, plasmid # 61245)—and used during flow cytometry and live-cell imaging. The excitation (ex)/emission (em) for pCMV G-CEPIA1er ex/em; 488 nm/512 nm, pCMV CEPIA2mt ex/em: 488 nm/500–550 nm, CMV-mito-LAR-GECO1.2 ex/em: 550–570 nm/590 nm.

### Live-cell imaging and microscopy

HT22 cells were co-transfected with pCMV G-CEPIA1er and CMV-mito-LAR-GECO1.2 for 48 h prior to seeding into Nunc Lab-Tek II chambered coverglass system (ThermoFisher; 155409) at 1 × 10^3^ cells/chamber and maintained in Ca^2+^-replete media overnight (Thermofisher; 11995). The following day, the cells were pretreated with 1 μM J147 for 1 h before the medium was removed and the cells were washed 3× with Ca^2+^-free HBSS without phenol red (ThermoFisher; 14175) supplemented with 0.1% FBS (ThermoFisher; 160000) immediately before the addition of thapsigargin (Cayman; 10522). Baseline fluorescence was recorded for 30 s prior to thapsigargin addition. 488 and 568 nm lasers were used to excite pCMV G-CEPIA1er and CMV-mito-LAR-GECO1.2, respectively. *Z*-stack images were acquired every 30 s and imaged on a Zeiss LSM 880 rear port laser scanning confocal and Airyscan FAST microscope. Zen Black and Imaris imaging analysis software were used to trace and render cells in 3D to quantitate changes in total cell fluorescence over time.

### Flow cytometry

Indo-1 was used to assess Ca^2+^ release into the cytosol from internal stores in real time in addition to measuring changes in Ca^2+^ content in HT22 cells transfected with either fluorescent Ca^2+^ indicators specific for the ER (pCMV G-CEPIA1er) or mitochondria (CEPIA2mt) during oxytosis, RSL3, and IAA toxicity time course experiments. To measure Ca^2+^ release from internal stores, HT22 cell were treated with 1 μM J147 or DMSO vehicle control overnight prior to staining with Indo-1. Cells were trypsinized, washed 3× with Ca^2+^-free HBSS supplemented with 0.1% FBS (ThermoFisher; 160000) and stored on ice. Cells were warmed to 37 °C immediately prior to measurement. Baseline recordings were made for 30–45 s before the addition of 3 μM thapsigargin. Ratiometric acquisition mode and kinetic parameter platform analysis in FlowJo (v10.5.3) were used to measure changes in cytosolic Ca^2+^ on a per cell basis. In toxicity time course experiments, glutamate (10 mM), RSL3 (500 nM), and IAA (12 μM) were used to assess Ca^2+^ content across the ER and mitochondrial compartments, while PI uptake was used as an index for cell viability. Indo-1 was used in combination with the Ca^2+^ re-addition method to assess SOCE^13^, where baseline measurements were recorded for 30 s followed by the addition of 3 μM thapsigargin to empty ER Ca^2+^ stores into the cytosol after which 3 mM CaCl_2_ was added back to allow for extracellular-derived Ca2^+^ influx.

### Reporting summary

Further information on experimental design is available in the Nature Research Reporting Summary linked to this article.

## Supplementary information

Supplementary Information

Reporting Summary

## Data Availability

The data that support the findings of this study are available from the corresponding author upon reasonable request. The data discussed in this publication have been deposited in NCBI’s Gene Expression Omnibus (Edgar, Domrachev, & Lash, 2002) and are accessible through GEO Series accession number GSE101112.
