# Supplementary material for: Targeting of intracellular Ca2+ stores as a therapeutic strategy against age-related neurotoxicities
Source: NPJ Aging Mech Dis. 2020 Aug 24;6:10. doi: 10.1038/s41514-020-00048-1 (PMC7445274; doi:10.1038/s41514-020-00048-1)
Supplement: Supplementary file 1 — Supplementary Information [file 41514_2020_48_MOESM1_ESM.pdf]

Supplementary Table 1

GOCC: Transporter complex_related to ATPase activity and Ca2+ channel	Subcellular Location	GOMF: Metal ion transmemebrane transporter activity	Subcellular Location
Kcna1	ER and PM	ATP12a	PM
Kcnq5	PM	Trpv2	PM, cytoplasm
Lrrc8b	ER and PM	Tusc3	ER, mitochondria,
Sacm1 l	ER; also golgi and PM	Kcnf1	PM, cytoplasm
Scn2a	PM	Atp2b4	PM
Gabrb2	PM	Kcnj9	PM
Atp2a2	ER and sarcoplasmic reticulum (SR)	Trpc1	PM
Cacna2d1	PM and SR	Kcna6	PM
Sestd1	Cytoskeleton	Nipal3	PM
Pde4d	Apical PM, cytoskeleton, nucleus	Grik3	PM
Scn1a	PM, nucleus	Slc8a3	ER, mitochondria, PM
Kcna2	ER, PM	Cacna1i	PM
Akap6	ER, nucleus, PM	Stim1	ER, PM, cytoskeleton, SR
Grin2a	ER, PM	Slc9a9	Endosome, PM
Kcnq3	PM	AtP1a1	PM, ER, endosome, golgi
Cacna1d	Nucleus,PM	Trpm4	ER, PM, golgi, cytosol, nucleus
Glra3	PM	Kcnk3	PM
Kcna3	PM	Kcns1	PM
Nlgn1	PM, golgi apparatus	Kcnk4	PM
Kcnc2	PM	Grin2b	PM
Stxbp5	PM, cytoplasm	Kcng3	ER, PM, cytoplasm
Pex5l	PM, peroxisomal membrane	Cacng7	PM, endosome
Scn8a	PM	Slc39a10	PM
Kcnb2	PM	Cacnb4	PM, nucleus, cytoplasm
Cacna1b	PM	Zdhhc17	PM, golgi, cytoplasm
Chrna6	PM	Kcnb1	PM, ER
Glra1	ER, PM	Kcnh5	PM
Cacna1g	PM	Slc30a4	Lysosome, endosome, PM
Pkd2l1	PM, ER	Scn1a	PM, nucleus
Ttyh2	PM	Cacng2	PM, cytosol, synaptosome
Cacng5	PM	Atp2b2	ER, PM
Kcnh2	PM, nucleus, cytoplasm	Kcnh1	PM, endosome, nucleus
Cntnap1	PM	Itgav	PM, cytosol
Kcnd1	PM	Lrrc55	PM

Cntn2	PM	Kcnv1	PM
Clic5	Golgi, cytoskeleton, nucleus	Tmc1	PM
Pkd1	ER, golgi, PM, nucleus	Kcna1	PM, ER
Lrrc8a	PM	Kcnq5	PM
Lrrc8c	ER, PM, cytoplasm	Atp2a2	ER, PM, nucleus
Shisa8	PM	Kcnt2	PM
Kcnq4	PM, cytoplasm	Scn2a	PM
Kcnc1	PM	Cacna2d1	ER, PM
Lrrtm4	PM	Kcnk9	PM
Amigo1	PM	Kcna2	ER , PM
Scn2b	PM	Grm7	PM, ER, golgi
Grin1	ER, PM	Nalcn	PM
Abcc8	PM	Kcna3	PM
Pex14	Nucleus, peroxisomal membrane	Slc9a7	PM, endosome, golgi
Pkd1l3	PM	Grin2a	ER, PM
Grin2d	PM	Kcnh7	PM
Vwc2l	PM	Kcnq3	PM
Scnn1b	PM	Slc1a6	PM, cytoskeleton, golgi
Pln	ER, mitochondria, SR	Kcnc2	PM
Gabrr2	PM	Kcnb2	PM
Glrb	ER, PM	Cacna1b	PM
Gabra3	PM	scn8a	PM
Olfm2	Nucleus, PM	Kcnq4	PM, cytoplasm
Dpp10	PM	Kcnc1	PM
Gria4	PM	Slc23a2	PM, cytoplasm
Lrrc55	PM	Slc4a10	PM
Cacnb4	Nucleus, PM, cytoplasm	Scn2b	PM
Olfm3	Golgi, PM	Slc9a6	ER, PM, endosome, mitochondria,
Stx1a	PM, cytoskeleton, nucleus	Slc6a17	PM, synaptic vesicle membrane
Grik3	PM	Gas6	Cytoplasm
Kcnf1	PM	Slc12a4	PM
Chrna4	PM	Slc5a6	PM
Cacng7	PM, endosome (early endosome)	Slc31a1	PM, endosome, cytoplasm
Cacna1i	PM	Slc13a4	PM
Kcnb1	ER, PM	Slc12a2	PM
Atp1a1	ER, PM, endosome, golgi	Slc6a20a	PM
Kcnk4	PM	Slc6a13	PM
Kcns1	PM	Slc13a3	PM
Kcna6	PM	Tfrc	PM, mitochondria, endosome, nucleus
Gabra4	PM	Slc12a5	PM

Kcnh1	Endosome, nuclear membrane, PM	Cacna1g	PM
Kcnv1	PM	Pkd2l1	ER, PM
Cacng2	PM, cytosol	Bzrap1	Mitochondria, cytoplasm
Vwc2	PM	Cacng5	PM
Trpm4	ER, PM, golgi, nucleoplasm	Kcnh2	PM, nucleus
Kcne4	PM	Trpm7	PM, cytoplasm
Gabrg2	PM	Nipa1	PM, endosome
Gabra1	PM	slc24a4	PM, cytoplasm
Trpc1	PM, SR	Mrs2	Mitochondria
Chrnb3	PM	Pkd1	ER, PM, golgi apparatus, nucleus
Grin2b	PM	Itpr3	ER, PM, nucleus
Kcng3	ER, PM, cytoplasm	Cacna1d	PM, nucleus
		Slc30a1	ER, PM, nucleus, golgi
		Atp1a4	PM
-	-	Trpa1	PM
		Cnnm4	PM
		Grin1	ER, PM
		Abcc8	Mitochondria, PM
		slc6a15	PM
		Slc39a6	ER, PM
		Kcnk12	PM
		Pkd1l3	PM
		Slc39a14	PM
		Erc1	Cytosol, golgi
		Slc39a9	PM
		Scnn1b	PM
		Trpv6	PM
		Slc39a5	PM
		Grin2d	PM
		Kcne4	PM
		Atp7b	ER, endosome, golgi
		Slc30a2	PM, endosome
		Kcnh8	PM
		Mmgt1	ER, PM, endosome, golgi, cytoplasm
		Trpm2	PM, lysosome
		Slc9a8	Golgi apparatus
		Slc6a1	PM
		Slc11a2	PM, mitochondria, nucleus, golgi, lysosome
		Kcnd1	PM
		Slc24a1	PM

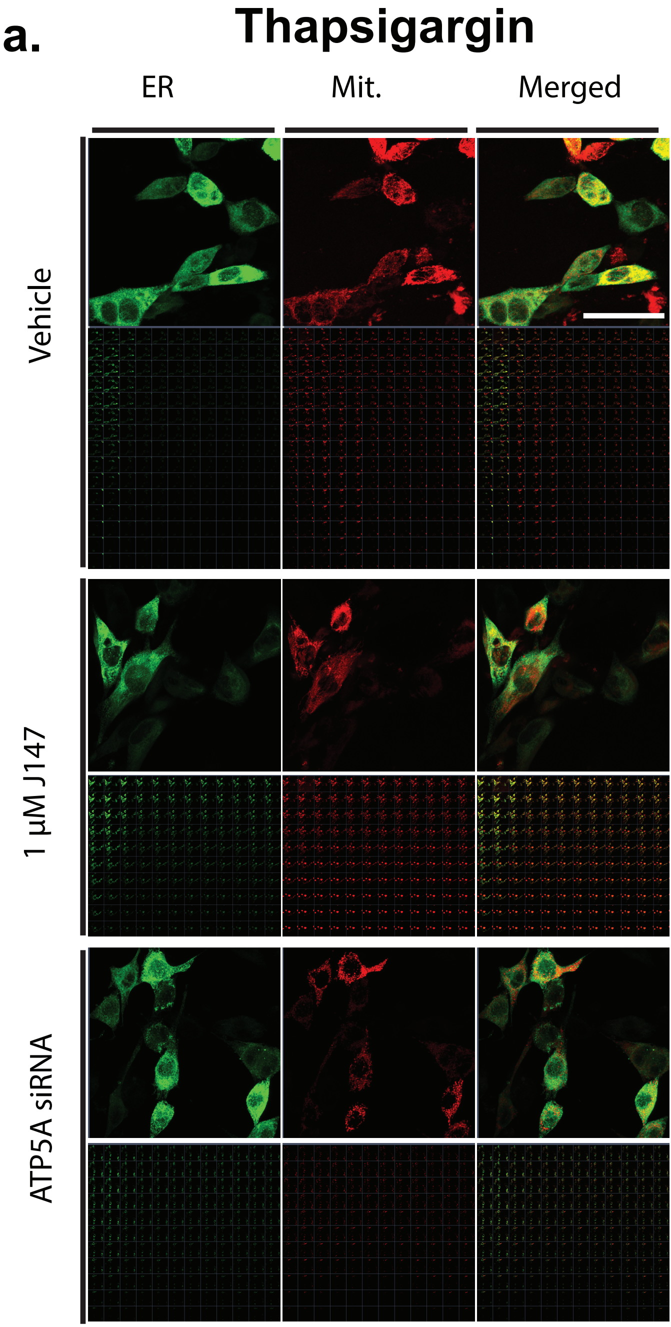
a.
Thapsigargin
ER
Mit.
Merged
Vehicle
1 μM J147
ATP5A siRNA

Supplementary Figure 1 Legend

J147 and ATP synthase knockdown buffer against ER Ca^{2+} release and transfer to mitochondria. HT22 cells treated with 1 μM J147 or ATP5A siRNA transfection were co-transfected with ER and mitochondrial-specific fluorescent Ca^{2+} sensors to monitor changes in fluorescence via Z-stack confocal microscopy live cell imaging after 5 μM thapsigargin treatment in Ca^{2+}-free media. Baseline fluorescence was recorded for 30 sec before addition of thapsigargin. Fold changes in fluorescence for J147 and ATP5A siRNA were recorded at 30 sec intervals and normalized to baseline fluorescence intensity for each condition in each treatment group. The Ca^{2+} sensors are ER *(green)* and mitochondria *(red)*. Second row of each drug/siRNA condition: Z-plane (y-axis), time (x-axis, 30 sec. intervals, $t_{=0}$ left-> t_{end} right).
