# Supplementary material for: Targeting of intracellular Ca2+ stores as a therapeutic strategy against age-related neurotoxicities
Source: NPJ Aging Mech Dis. 2020 Aug 24;6:10. doi: 10.1038/s41514-020-00048-1 (PMC7445274; doi:10.1038/s41514-020-00048-1)
Supplement: Supplementary file 2 — Reporting Summary [file 41514_2020_48_MOESM2_ESM.pdf]

nature research

Corresponding author(s): Pamela Maher

Last updated by author(s): Jul 27, 2020

Reporting Summary

Nature Research wishes to improve the reproducibility of the work that we publish. This form provides structure for consistency and transparency in reporting. For further information on Nature Research policies, see our Editorial Policies and the Editorial Policy Checklist.

Statistics

For all statistical analyses, confirm that the following items are present in the figure legend, table legend, main text, or Methods section.

n/a	Confirmed	
☐	☒	The exact sample size (*n*) for each experimental group/condition, given as a discrete number and unit of measurement
☐	☒	A statement on whether measurements were taken from distinct samples or whether the same sample was measured repeatedly
☐	☒	The statistical test(s) used AND whether they are one- or two-sided<br>*Only common tests should be described solely by name; describe more complex techniques in the Methods section.*
☒	☐	A description of all covariates tested
☒	☐	A description of any assumptions or corrections, such as tests of normality and adjustment for multiple comparisons
☐	☒	A full description of the statistical parameters including central tendency (e.g. means) or other basic estimates (e.g. regression coefficient) AND variation (e.g. standard deviation) or associated estimates of uncertainty (e.g. confidence intervals)
☐	☒	For null hypothesis testing, the test statistic (e.g. *F*, *t*, *r*) with confidence intervals, effect sizes, degrees of freedom and *P* value noted<br>*Give P values as exact values whenever suitable.*
☒	☐	For Bayesian analysis, information on the choice of priors and Markov chain Monte Carlo settings
☒	☐	For hierarchical and complex designs, identification of the appropriate level for tests and full reporting of outcomes
☐	☒	Estimates of effect sizes (e.g. Cohen's *d*, Pearson's *r*), indicating how they were calculated

Our web collection on statistics for biologists contains articles on many of the points above.

Software and code

Policy information about availability of computer code

Data collection	*Provide a description of all commercial, open source and custom code used to collect the data in this study, specifying the version used OR state that no software was used.*
Data analysis	Reads generated by the sequencer (PE 100) were mapped to the mouse transcriptome using the spliced aligner STAR (2.5.1b) with default parameters. Only uniquely aligned reads are considered for downstream analysis. Expression values were quantified using Homer (4.9.1 [59]) by counting reads mapped across all gene exons of RefSeq genes and mitochondrial encoded genes. The differential expression analysis was performed by edgeR (v3.16.1). Gene set enrichment analysis was performed using WebGestaltR.

For manuscripts utilizing custom algorithms or software that are central to the research but not yet described in published literature, software must be made available to editors and reviewers. We strongly encourage code deposition in a community repository (e.g. GitHub). See the Nature Research guidelines for submitting code & software for further information.

Data

Policy information about availability of data

All manuscripts must include a data availability statement. This statement should provide the following information, where applicable:

- Accession codes, unique identifiers, or web links for publicly available datasets
- A list of figures that have associated raw data
- A description of any restrictions on data availability

The data that support the findings of this study are available from the corresponding author upon reasonable request. The data discussed in this publication have been deposited in NCBI's Gene Expression Omnibus (Edgar, Domrachev, & Lash, 2002) and are accessible through GEO Series accession number GSE101112 (https://www.ncbi.nlm.nih.gov/geo/query/acc.cgi?acc=GSE101112).

Field-specific reporting

Please select the one below that is the best fit for your research. If you are not sure, read the appropriate sections before making your selection.

- [x] Life sciences
- [ ] Behavioural & social sciences
- [ ] Ecological, evolutionary & environmental sciences

For a reference copy of the document with all sections, see nature.com/documents/nr-reporting-summary-flat.pdf

Life sciences study design

All studies must disclose on these points even when the disclosure is negative.

Sample size	For the animal studies, the sample size was based on prior studies with this strain of mice and the types of analyses that we planned to do. For the cell culture studies, sample size was based on our previous experience with these types of assays.
Data exclusions	No data was excluded,
Replication	All cell culture experiments were repeated at least times.
Randomization	Mice were randomly assigned to the different groups.
Blinding	Analysis of the mice was not blinded because we needed to know which group each mouse belonged to in order to do the analyses.

Reporting for specific materials, systems and methods

We require information from authors about some types of materials, experimental systems and methods used in many studies. Here, indicate whether each material, system or method listed is relevant to your study. If you are not sure if a list item applies to your research, read the appropriate section before selecting a response.

Materials & experimental systems

n/a	Involved in the study	
x		Antibodies
	x	Eukaryotic cell lines
x		Palaeontology and archaeology
	x	Animals and other organisms
x		Human research participants
x		Clinical data
x		Dual use research of concern

Methods

n/a	Involved in the study	
x		ChIP-seq
	x	Flow cytometry
x		MRI-based neuroimaging

Eukaryotic cell lines

Policy information about cell lines

Cell line source(s)	The HT22 cells were developed in our laboratory. We have sent them to many labs around the world and they can also be purchased from Millipore.
Authentication	The HT22 cells are authenticated based on their sensitivity to the toxins used in this study.
Mycoplasma contamination	No evidence of mycoplasma contamination was found the last time we checked.
Commonly misidentified lines (See ICLAC register)	none

Animals and other organisms

Policy information about studies involving animals; ARRIVE guidelines recommended for reporting animal research

Laboratory animals	SAMP8 mice
Wild animals	*Provide details on animals observed in or captured in the field; report species, sex and age where possible. Describe how animals were caught and transported and what happened to captive animals after the study (if killed, explain why and describe method; if released, say where and when) OR state that the study did not involve wild animals.*
Field-collected samples	*For laboratory work with field-collected samples, describe all relevant parameters such as housing, maintenance, temperature, photoperiod and end-of-experiment protocol OR state that the study did not involve samples collected from the field.*

Ethics oversight	All experiments were performed in accordance with the US Public Health Service Guide for Care and Use of Laboratory Animals and the protocols were approved by the IACUC at the Salk Institute.

Note that full information on the approval of the study protocol must also be provided in the manuscript.

Flow Cytometry

Plots

Confirm that:

- [x] The axis labels state the marker and fluorochrome used (e.g. CD4-FITC).
- [x] The axis scales are clearly visible. Include numbers along axes only for bottom left plot of group (a 'group' is an analysis of identical markers).
- [ ] All plots are contour plots with outliers or pseudocolor plots.
- [x] A numerical value for number of cells or percentage (with statistics) is provided.

Methodology

Sample preparation	HT22 cells were trypsinized, centrifuged at 500 x g, and resuspended in HBSS and stored on ice. For Ca2+ flux analysis, samples were warmed to 37 degrees Celcius prior to agonist addition.
Instrument	BD LSR II
Software	FloJo 10.5.3
Cell population abundance	Post-sorted fractions were not used for analysis in this manuscript.
Gating strategy	Antibody staining was not used in this manuscript, only fluorescent calcium indicator dyes and kinetic flux analysis. Thus, entire populations were used for this type of analysis and gating strategy is not relevant.

- [ ] Tick this box to confirm that a figure exemplifying the gating strategy is provided in the Supplementary Information.
